# Magnetic Resonance Imaging of Musculoskeletal Manifestations in Sickle Cell Disease

**DOI:** 10.3390/jcm14228056

**Published:** 2025-11-13

**Authors:** Jaber Hussain Alsalah

**Affiliations:** 1 Department of Radiological Sciences, Faculty of Applied Medical Sciences, King Abdulaziz University, Jeddah 21589, Saudi Arabia; jhalyami@kau.edu.sa; 2 Smart Medical Imaging Research Group, King Abdulaziz University, Jeddah 21589, Saudi Arabia; 3 King Fahd Medical Research Center, King Abdulaziz University, Jeddah 21589, Saudi Arabia

**Keywords:** MSK MRI, sickle cell disease, musculoskeletal manifestations, imaging patterns

## Abstract

**Background**: Sickle cell disease (SCD) affects more than 100,000 people in the United States and 8 million people worldwide, with high morbidity and mortality and musculoskeletal (MSK) complications that contribute to functional disability. However, MRI-based characterization of musculoskeletal manifestations remains limited in high-prevalence regions, including the Middle East. This study aimed to review MRI findings of MSK manifestations in SCD patients and assess associations with clinical characteristics. **Methods**: A retrospective study was conducted on 96 patients with SCD who underwent MSK MRI between 2012 and 2022 at King Abdulaziz University Hospital. Patient demographics, clinical characteristics, and imaging findings were reviewed. The prevalence and distribution of MSK complications were analyzed across age, gender, and BMI categories. **Results**: Of the 96 patients (47% males; 53% females; mean age 28.9 years), the hip was the most frequently scanned region (46%), followed by the leg, femur, shoulder, and knee. Bone infarction was the most common complication, observed in 57 patients (59.3%), and was more prevalent among older adults. Osteomyelitis was identified in 16 patients (16.7%), with higher rates in children and underweight individuals. Decreased bone marrow signal intensity was seen in 11 patients (11.4%), particularly in older age groups. Other findings and unremarkable scans each accounted for 6 cases (6.3%). Gender analysis showed broadly similar patterns, although decreased marrow signal intensity was more common in females. **Conclusions**: MRI is an effective imaging modality for detecting and differentiating MSK complications in SCD. Routine use of MRI in follow-up care is recommended to facilitate early diagnosis, guide management, and prevent long-term disability. Larger prospective studies are needed to validate these findings and establish MRI as a routine diagnostic tool for SCD.

## 1. Background

Sickle cell anemia (SCA) is an autosomal recessive genetic blood disorder characterized by red blood cells that assume an abnormal, rigid, and sickle shape. These cells have reduced deformability, leading to chronic hemolysis, vaso-occlusion, and an increased risk of systemic complications, including musculoskeletal complications such as osteonecrosis, osteomyelitis, osteoporosis, and bone fractures, affecting 50–70% of SCD patients [1]. SCD is one of the most common hereditary hemoglobinopathies worldwide. However, MRI-based characterization of musculoskeletal manifestations remains limited in high-prevalence regions, including the Middle East, where disease burden and presentation patterns may differ [2,3,4]. SCD has a high prevalence in Africa, where more than 200,000 affected infants are born annually, and affects about 72,000 individuals in the United States, with over 2 million carriers. In Saudi Arabia, SCD was first identified in the Eastern Province in the 1960s, which led to regional and national screening studies to determine its frequency and clinical characteristics [2]. Current premarital and newborn screening initiatives report carrier rates of 21% and disease prevalence of 2.6%, with consanguinity rates exceeding 50% contributing to its persistence [3,4].

Musculoskeletal complications are common in sickle cell disease (SCD) and contribute significantly to both acute and chronic morbidity [5]. Acute problems include vaso-occlusive crises with bone infarcts and osteomyelitis, while chronic complications include osteoporosis and osteonecrosis, particularly avascular necrosis of the femoral and humeral heads. These result from red blood cell sickling, which causes hypoxia, ischemia, and blood stasis in the bone marrow [1,6]. Anemia-related changes, such as extramedullary hematopoiesis, bone expansion, and pathologic fractures, are also common. Furthermore, vaso-occlusion leads to growth disturbances, H-shaped vertebrae, dactylitis, septic arthritis, and osteomyelitis, while severe ischemia may cause muscle necrosis, soft tissue hematomas, and abscess formation [1,4,5,6]

Conventional radiography remains the first-line investigation for musculoskeletal complaints but has poor sensitivity and specificity, with changes often lagging behind histopathological bone alterations by up to two weeks [7,8,9]. Consequently, poor sensitivity for early detection, poor specificity of findings, often in the setting of underlying chronic changes such as osteonecrosis and arthritis, and equivocal radiographic findings often prompt further evaluation with MRI [10,11,12,13,14].

Consequently, MRI is the preferred method for evaluation, as it offers the best balance of sensitivity and specificity and allows for the early detection of osseous changes [15]. MRI is superior to the other imaging modalities in detecting bone marrow lesions [16]. Primary MR findings of osteomyelitis include decreased marrow signal on T1-weighted images, increased signal on T2-weighted images, and enhancement on post-contrast T1-weighted imaging. MRI is also useful for showing adjacent soft tissue fluid collections, cellulitis, cortical bone interruption, sinus tracts, and possible sequestra [17]. Such detailed visualization enables a more precise evaluation of severity, prognosis, treatment selection, and outcomes [14].

Despite the recognized value of MRI in SCD, relatively few studies have comprehensively characterized the full musculoskeletal spectrum across skeletal regions or correlated imaging with demographics and clinical features in high-prevalence regions, including Saudi Arabia and the Middle East. This study, therefore, aims to determine musculoskeletal (MSK) manifestations in patients with sickle cell disease (SCD) at King Abdulaziz University Hospital (KAUH) and to assess the relationship between these findings and demographic and clinical characteristics. Additionally, the study seeks to identify common MSK complications and assess their prevalence across age, gender, and BMI groups. By providing population-specific evidence, this work may enhance understanding of SCD complications within settings where genetic and consanguinity factors contribute to higher disease prevalence. The findings may also support improved diagnostic accuracy and inform management strategies for this challenging disease.

## 2. Material and Methods

### 2.1. Study Design and Participants

This was a retrospective study including 96 patients with confirmed sickle cell disease who underwent clinically indicated musculoskeletal MRI between 2012 and 2021 at King Abdulaziz University Hospital. The study received ethical approval from the Research Ethics Committee (REC), Jeddah, Saudi Arabia on 18 September 2022, with the registration number (HA-02-J-008). Inclusion criteria were patients with a confirmed SCD diagnosis who underwent musculoskeletal MRI for clinical evaluation and had reports in the system. MRI cases of SCD patients with incomplete clinical or demographic data or missing MRI reports, and MRI studies with non-diagnostic image quality, were also excluded.

#### Data Collection

Demographic and clinical data for SCD patients were collected, including age, gender, nationality, blood group, Hb and HCT levels, and BMI categories. MSK MRI findings were retrospectively extracted from electronic medical records.

### 2.2. MRI Acquisition

The MRI data were acquired from 3-T and 1.5-T MRI scanners at King Abdul-Aziz University Hospital. The MSK MRI protocols used sequences for common orthopedic indications (Appendix A).

### 2.3. Statistical Analysis

Descriptive analysis was performed using SPSS v26 software. Age was expressed as the mean and standard deviation. Categorical variables were expressed as counts and percentages. Frequencies and percentages are reported to present the overall patterns. All data are presented in tabular and graphical formats.

## 3. Results

### 3.1. Patient Characteristics

The study included 96 patients with musculoskeletal complications; patient characteristics are summarized in Table 1. The mean age was 28.3 ± 13.0 years, with 47.9% of patients aged between 20 and 39 years. Females accounted for 53.1% (51/96) and males for 46.9% (45/96). The majority were Saudi nationals (61.5%), followed by Yemenis (31.3%) and others (7.3%), mainly of African or Arab origin. The mean body mass index (BMI) was 21.3 ± 5.8, with 34.4% of patients underweight, 44.8% normal, 11.5% overweight, and 9.4% obese.

The clinical characteristics of patients with MSK complications showed that blood group O+ (57%) was the most frequent, followed by A+ (23%), B+ (11%), and AB+ (8%). The mean hemoglobin level was 8.45 ± 1.57 g/dL, and the mean hematocrit level was 24.71 ± 4.78% (Table 2).

### 3.2. MSK Scanned Parts

**Figure 1:**
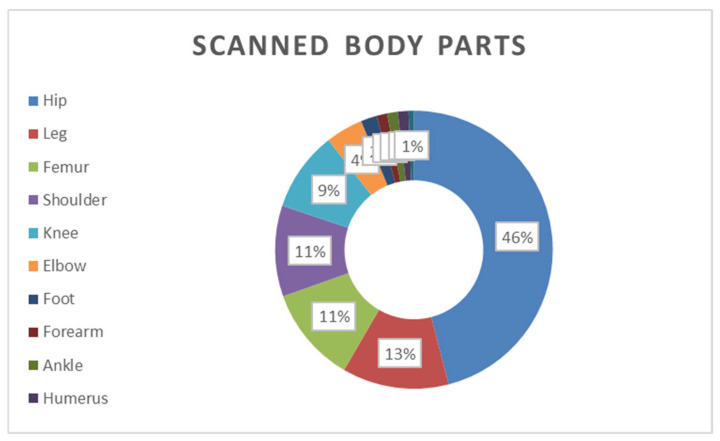
Scanned parts of musculoskeletal regions (n = 63).

A total of 163 MRI scans were performed on 96 patients with musculoskeletal (MSK) complications. As shown in Figure 1, the hip was the most frequently scanned region (46%), followed by the leg (13%), femur (11%), shoulder (11%), and knee (9%). Other regions of the scans included the foot, forearm, ankle, humerus, and hand, which accounted for 22%.

### 3.3. MRI Indications in Patients with MSK Complications

Among the 96 patients with musculoskeletal (MSK) complications, the most common MRI indication was pain with suspicion of avascular necrosis (AVN), accounting for 57% of referrals. This was followed by pain with suspicion of osteomyelitis in 37% of cases. The remaining 6% of referrals were attributed to other indications.

### 3.4. MRI Findings in Patients with MSK Complications

**Figure 2:**
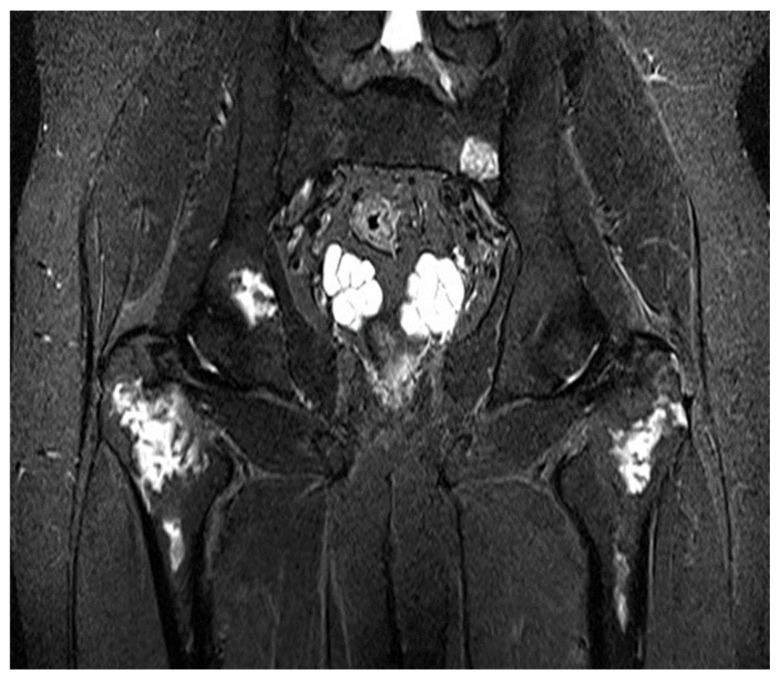
Coronal STIR MRI of a 27-year-old male patient with multiple areas of osteonecrosis and bilateral femoral head avascular necrosis.

MRIs of patients with musculoskeletal (MSK) complications revealed that the most frequent abnormality was bone infarction, identified in 59% (57 of the 96 patients). Bone infarction was common among adults (73.8%), males (72.6%), and obese patients (90%) (Figure 2).

**Figure 3:**
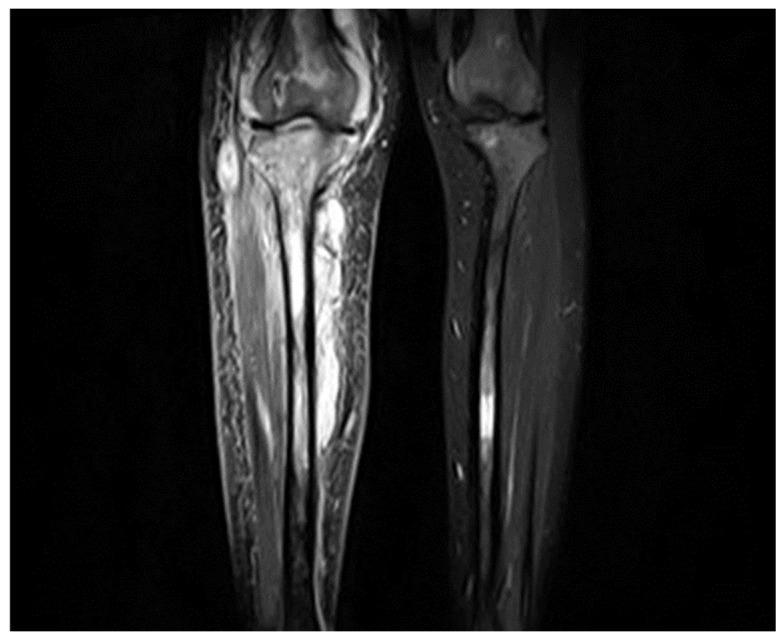
Coronal T1-weighted MR image of a 26-year-old female with fluid collection surrounding the tibia with underlying bone changes indicating tibial osteomyelitis.

Osteomyelitis was the second most common NSK complication, occurring in 17% of patients. Children had the highest rate (35%) compared to adults, and underweight patients were particularly affected (31.6%) (Figure 3).

**Figure 4:**
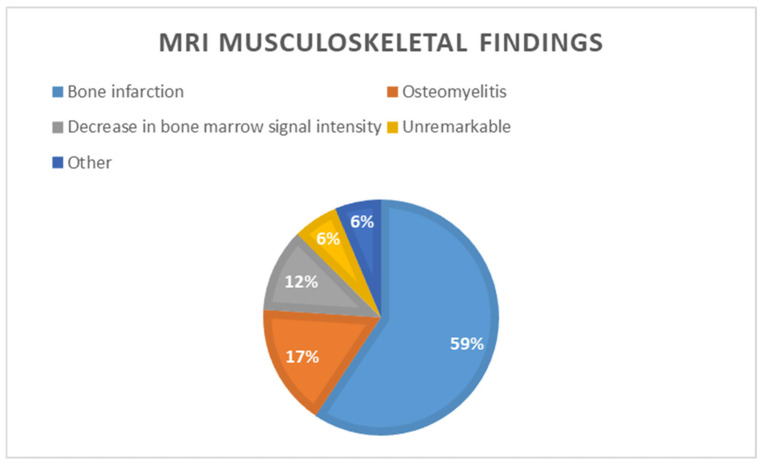
Distribution of MRI musculoskeletal findings in the study.

A decreased bone marrow signal intensity was observed in 12% of the patients. The remaining scans were either classified as miscellaneous other findings or showed no remarkable abnormalities (Figure 4).

Bone infarction was the most common abnormality and was more frequently observed in adults, whereas osteomyelitis occurred predominantly in children. As shown in Table 3, bone infarction was observed in 57 patients (59.3%), predominantly in those aged ≥36 years. Osteomyelitis occurred in 16 patients (16.7%), mainly between 6 and 15 years and 16 and 20 years. Decreased bone marrow signal intensity was more prominent in older patients; it was found in 11 patients (11.4%), particularly in older age groups, while other complications and unremarkable findings were each noted in 6 patients (6.3%). According to Table 3, bone infarction was the leading complication in both females (52.9%) and males (66.7%). Decreased bone marrow signal intensity was more common in females, whereas other complications were slightly more frequent in males.

## 4. Discussion

This study reviews musculoskeletal findings in patients with sickle cell disease (SCD) at KAUH and provides region-specific MRI findings for Saudi Arabia. Bone infarction was the most frequent MRI finding in our cohort, particularly among adults, whereas osteomyelitis occurred more often in children. These findings are consistent with earlier studies, which reported multiple bone infarctions in nearly all patients [18]. The present study also showed that bone infarction predominantly affected the long bones and axial skeleton. According to Kosaraju et al. [4], the long bones are the most frequent sites of infarction, with osteonecrosis involving the epiphyseal regions of long bones commonly referred to as avascular necrosis (AVN). The femoral and humeral heads are the most commonly affected sites, which aligns with our observations [4].

The second most common musculoskeletal complication was osteomyelitis, with a prevalence of 17%. Patients with SCD are more susceptible to osteomyelitis due to several mechanisms, including hyposplenism, impaired complement activity, and the presence of infarcted or necrotic bone [19,20,21]. Almeida et al. (2005) also reported osteomyelitis in a French study of a cohort of 299 patients followed in four Parisian centers, with a prevalence of 12% [20]. These findings highlight the importance of early recognition and multidisciplinary management to reduce long-term morbidity. Osteomyelitis usually affects the diaphysis of long bones, though other sites, such as vertebrae, can also be involved [21]. Clinically, patients may present with fever, restricted mobility, tenderness, and swelling of affected regions [17]. Early and accurate diagnosis is critical, as untreated osteomyelitis can lead to bone destruction and deformity. Importantly, clinical and radiographic features of acute osteomyelitis may be difficult to distinguish from bone infarction; in this context, magnetic resonance imaging (MRI) is particularly valuable. MRI findings such as cortical defects, adjacent soft tissue collections, and bone marrow enhancement are suggestive of infection [15,16,17,18].

Comparisons with studies from other regions reveal similar patterns. A study conducted in Hail, Saudi Arabia, on children with SCD reported AVN and osteomyelitis as the major musculoskeletal complications, followed by bone infarction. Similarly, a retrospective study from Brazil reported AVN and osteomyelitis as the most frequent findings. In contrast, a study from Nigeria on children with SCD demonstrated that acute infarction was the most common musculoskeletal manifestation [22]. An Egyptian study identified vertebral bone infarctions as the predominant finding, followed by femoral head AVN, with osteomyelitis and septic arthritis being less frequent [18]. Another report highlighted vaso-occlusive crisis as the most common bone pathology in SCD patients, followed by osteomyelitis, with the incidence of acute osteomyelitis significantly higher in SCD patients compared to the general population [22,23]. Additionally, a recent systematic review on SCD in Saudi Arabia reported that the most frequently documented complications included vaso-occlusive crises, acute chest syndrome, splenic sequestration, osteomyelitis, and renal involvement, along with recurrent painful crises and infectious complications [24].

The limitations of this study include its retrospective design, single-center nature, and use of incomplete medical records, which limited the comprehensiveness of the data. Additionally, the small sample size reduced the study’s statistical power. A larger sample size and a prospective study design would help clarify these associations and yield more robust conclusions.

## 5. Conclusions

Sickle cell disease (SCD) is a significant health problem associated with high morbidity and mortality, substantially contributing to the burden of musculoskeletal complications. In this study, bone infarction was the most common musculoskeletal abnormality, followed by osteomyelitis and decreased bone marrow signal intensity. Bone infarction was more prevalent in older adults, while osteomyelitis was observed more frequently in children and underweight patients, highlighting variation across age and body composition. Gender-based differences were minimal, though decreased marrow signal intensity was more common in females. The hip was the most frequently affected region, and suspicion of avascular necrosis was the leading MRI indication. These findings emphasize the critical role of MRI in the early detection and monitoring of skeletal changes in SCD. Further prospective multicenter research is recommended. MRI should be utilized when clinically indicated to support early detection of musculoskeletal complications and guide appropriate management strategies. This may enable earlier recognition of complications, reduce long-term disability, and ultimately improve patients’ quality of life.

## Figures and Tables

**Table 1:** Patient characteristics.

Variable	Categories	Patients (n = 96)
Age (M = 28.32; SD = 13.01)	<10	7 (7.29%)
	10–19	21 (21.88%)
	20–29	22 (22.92%)
	30–39	24 (25%)
	40–49	19 (19.79%)
	50+	3 (3.13%)
Gender	Male	45 (46.88%)
	Female	51 (53.13%)
BMI (M = 21.34; SD = 5.84)	Underweight	33 (34.38%)
	Normal	43 (44.79%)
	Overweight	11 (11.46%)
	Obese	9 (9.38%)
Nationality	Saudi	59 (61.46%)
	Yemeni	30 (31.25%)
	Other	7 (7.29%)

**Table 2:** Patient clinical characteristics.

Variable	Categories	Patients (n = 96)
Blood group	A	22 (22.92%)
	AB	8 (8.33%)
	B	11 (11.46%)
	O	55 (57.29%)
Hb Level (M = 8.45; SD = 1.57)	<8	30 (31.25%)
	8–10	54 (56.25%)
	>10	12 (12.5%)
HCT Level (M = 24.71; SD = 4.78)	<17	3 (3.13%)
	17–29	78 (81.25%)
	>29	15 (15.63%)

**Table 3:** Comparison of musculoskeletal complication patterns stratified by age categories and gender, demonstrating the frequency distribution of each imaging finding within the cohort (N = 96).

Complication	<5	6–10	11–15	16–20	21–25	26–30	31–35	36+	Total	Female	Male	Total
Bone infarction	1	1	4	8	6	8	7	22	57	27	30	57
Decrease in bone marrow signal intensity	0	0	2	0	0	0	3	6	11	9	2	11
Osteomyelitis	1	3	4	5	1	1	0	1	16	9	7	16
Others	0	0	0	1	0	2	0	3	6	2	4	6
Unremarkable	0	2	0	1	2	0	0	1	6	4	2	6
Total	2	6	10	15	9	11	10	33	96	51	45	96

## Data Availability

The original contributions presented in this study are included in the article. Further inquiries can be directed to the corresponding author.

## Appendix A

**Table A1:** Hip MRI protocol at King Abdulaziz University Hospital.

Sequence Name	TR (ms)	TE (ms)	Slice (mm)	Flip Angle (°)	Acquisition Time (min)
T1-TSE-COR-384	551	17–18	3.0	**70–90°**	**2.5–4.0**
T1-TIRM-COR-320 (STIR)	6290	28	3.0	**150°**	**3.5–5.0**
PD-TSE-COR	4230	33	3.0	**150°**	**3.0–4.5**
PD-TSE-TRA-FS	4430	30	3.0	**150°**	**3.5–5.0**
T1-SE-TRA	616	15	3.0	**70–90°**	**2.0–3.0**
PD-TSE-FS-SAG	3820	24	3.0	**150°**	**3.5–5.0**

**Table A2:** Leg MRI protocol at King Abdulaziz University Hospital.

Sequence Name	TR (ms)	TE (ms)	Slice (mm)	Flip Angle (°)	Acquisition Time (min)
T1-TIRM COR (STIR)	5000	29	4.0	**150°**	**3.0–4.0**
PD-TSE-FS COR (Fat-sat PD)	3000	37	4.0	**150°**	**3.5–5.0**
COR T1 Fat Sat	574	17	4.0	**70–90°**	**2.5–4.0**
T2-TSE SAG	4500	85	4.0	**150°**	**3.0–4.5**
AX PD Fat Sat	3270	38	4.0	**150°**	**3.5–5.0**
AX T1	822	11	4.0	**70–90°**	**2.0–3.0**
SAG PD Fat Sat	3000	28	4.0	**150°**	**3.5–5.0**
